# Leisure Activities of Healthy Children and Adolescents

**DOI:** 10.3390/ijerph16122078

**Published:** 2019-06-12

**Authors:** Lea Auhuber, Mandy Vogel, Nico Grafe, Wieland Kiess, Tanja Poulain

**Affiliations:** 1 LIFE Leipzig Research Center for Civilization Diseases, Leipzig University, Philipp-Rosenthal-Strasse 27, 04103 Leipzig, Germany; lea.auhuber@t-online.de (L.A.); mvogel@life.uni-leipzig.de (M.V.); nico.grafe@life.uni-leipzig.de (N.G.); wieland.kiess@medizin.uni-leipzig.de (W.K.); 2 Department of Women and Child Health, Hospital for Children and Adolescents and Center for Pediatric Research (CPL), Leipzig University, Liebigstrasse 20a, 04103 Leipzig, Germany

**Keywords:** leisure activities, media, physical activity, adolescents

## Abstract

The objective of the present study was to give a detailed overview on the leisure behavior of adolescents (frequency, differences between gender, age groups and social class, time trends, and inter-relations). In total, 1449 10- to 18-year-old German adolescents were included in the study. Participants answered questionnaires about their media use, physical activity, outdoor time, engagement in choir/orchestra and theater/dancing, social life and socio-economic status (SES). The results revealed that girls, children with lower SES as well as older children reported to use screen-based media more often and that girls, older children and children with lower SES were less physically active. In addition, boys and children with lower SES engaged less frequently in choir/orchestra and theater/dancing, while children with higher SES met their friends more often. The time trend analysis showed that mobile phone use increased drastically from 2011 to 2017, while engagement in choir/orchestra and theater/dancing decreased. Regarding the inter-relation between leisure activities, high screen times were significantly associated with less physical activity and less outdoor time. Physical activity, in contrast, was significantly related to better social life and more outdoor time. These findings highlight the growing importance of electronic media in adolescents’ lives and their tendency to displace other leisure activities.

## 1. Introduction

How someone spends his/her free time as a child (e.g., by being socially or physically active or inactive) may affect the leisure activities in adulthood. Children and adolescents who have been active in a variety of physical activities are also much more active as adults [1,2]. Furthermore, leisure activities can go along with an increased or decreased risk to develop diseases. For example, sufficient physical activity has been shown to contribute to physical and mental health [3], whereas excessive media use was reported to be associated with poorer mental and physical health [4]. For this reason, it is important to investigate the leisure activities of healthy children and adolescents and, if necessary, to intervene at an early stage.

The use of screen-based media plays an increasing role in the leisure activities of children and adolescents. Time trend analyses showed that the time children use electronic media, especially computers and mobile phones [5], has increased significantly in recent years [6,7]. With the increasing use of media, several guidelines (e.g., the Canadian Sedentary Behaviour Guidelines, the recommendation of the American Academy of Pediatrics, Austrian Society of Pediatrics and Adolescent Medicine) have been put into place to avoid excessive media use in childhood. Overall, these guidelines recommend a total daily media use of not more than two hours, with shorter periods benefiting health [8,9,10]. On weekdays, about one third of the young people in Germany were shown to exceed the recommendations just by watching television [11]. In general, boys spent more time using media (especially television, computer, and video games) than girls [7,11]. Moreover, older children and adolescents as well as children with lower socio-economic status (SES) were reported to spend more time using media [12,13,14].

Physical activity represents another important leisure activity of children and adolescents, which, in contrast to media use, remained nearly unchanged during the last years [5,15]. In Germany, a majority of children claimed to do sports, almost two third of them more than two hours a week [16]. Furthermore, nearly two thirds of German children reported being a member of a sports club, with boys being more active than girls [13]. Similar to media use, physical activity differs depending on social class, child age, and gender. Children from families with lower SES have been shown to be less active than children with a higher social status [13,16]. Furthermore, physical activity was reported to decrease with growing child age [17,18]. With respect to gender, girls were observed to be more active in individual sports but less engaged in team sports [19].

Many children and adolescents also spend their leisure time with activities other than media use and physical activity, e.g., music, arts, or social activities. In contrast to research on media use and physical activity, studies on these leisure activities are sparse. In studies conducted in Canada and in the Czech Republic, more than 80% of the children were shown to engage in at least one extracurricular activity [20,21]. Furthermore, girls were reported to be more likely to participate in artistic activities than boys [19]. Concerning social activities, almost three-quarters of German 12- to 19-year-old children were shown to meet regularly with friends, with boys and children from lower social status being more likely to go out with friends [22].

Most previous studies focused on one leisure activity only. However, several studies also investigated inter-relations between different leisure activities, especially between media use and physical activity. There is some evidence that physical activity has a direct negative relationship to inactive media use, namely TV/movie use [23] and that very high levels of media use are associated with lower physical activity [24]. However, it was also observed that media use and physical activity coexist rather than compete, especially in boys [25,26,27]. Friendship also plays an important role in the activity behavior of children and young people. Children whose friends are physically active were shown to be also more active themselves [28,29,30]. Whether the circle of friends also has an influence on media use is controversial [31].

Despite intensive research in the leisure behavior of children and adolescents, some research gaps remain. For example, it is nearly unexplored how often children and adolescents engage in leisure activities related to music or arts, how the popularity of these activities changed in the last years, and how they relate to more prominent activities such as media use and physical activity.

The present study provides an overview on the frequency of a variety of leisure activities in healthy children, including not only media use and physical activity but also social life (meeting friends), outdoor activity, choir/orchestra, and theater/dancing. The focus is put on differences in these activities depending on gender, age, and SES, on time trends (2011–2017), and on inter-relations between these activities. The results might indicate whether certain groups of children differ in their leisure behavior and whether certain leisure activities may favor or displace other activities. Finally, harmful combinations of leisure behaviors (e.g., high media use coupled with few other activities) might be detected in order to pave the way for interventions.

According to the previous state of research [5,6,7,24,25,30,31], the use of screen-based media was hypothesized to have increased in the last years and to go along with less physical activity, less outdoor activity, and also fewer other leisure activities (social contacts, engagement in choir/orchestra and theatre/dancing). Physical activity, in contrast, was expected to be related to a higher frequency of other leisure activities (except media use).

## 2. Materials and Methods 

### 2.1. Participants

The data used for the subsequent analysis were collected between 2011 and 2017 in the LIFE Child study center. LIFE Child is a longitudinal childhood cohort study located in Leipzig, Germany. The intention of this study is to research civilization diseases in childhood and the development of healthy children [32,33].

All 10- to 18-year-old children and adolescents who participated in LIFE Child and answered the questionnaires about their screen time, other leisure activities and socio-economic status (SES), were included. For participants who attended several times, only the last visit was selected. In total, 1449 children and adolescents (717 male, 732 female) with an average age of 14.2 (SD = 2.20) could be included in the present analyses.

A SES composite score (the so-called Winkler index) was employed to examine the socio-economic status of the participating children. The index contains information about the school-leaving qualifications and professional education, the current working situation, and the income of parents [34]. The score ranges between 3 and 21, with higher scores indicating higher SES. In a large representative German cohort study, Lampert et al. determined a distribution-based delimitation of the index into 5 equal groups (quintiles); 20% each belong to the lowest and the highest social class, the remaining 60% form the middle class [34]. In the present study, 15% of the participants belonged to the lower social class, while 59% were from the middle social class and 26% from the higher social class. Accordingly, the distribution of social classes in our sample is good, except for a slight tendency towards the higher social class.

Informed written consent was received from all participants over the ages of 18. In order for minors to participate in the study, the parents of the underage participants gave their written consent. Furthermore, children aged 12 and older consented additionally. The Ethics Committee of the Medical Faculty of the University of Leipzig approved the study (Reg. No. 264-10-19042010).

### 2.2. Measures

Screen-based media use, physical activity, outdoor activity, social life (meeting friends), and engagement in choir/orchestra and theatre/dancing as the major leisure activities of children and adolescents were assessed via questionnaires. Our analyses are based on children’s self-reports as we consider that children aged 10 years or older are able to estimate how much time they spend with different leisure activities. Parents, in contrast, might not always know what children do in their free time or tend to answer questions in socially desirable ways. The questionnaires were adapted to the survey used in German Health Interview and Examination Survey for Children and Adolescents (KiGGS), a nationwide study on the health of children and adolescents [13,35]. In the questionnaire on media use, participants were asked how much time a day they spend with screen-based media, including TV, game consoles, PC and mobile phone. The participants were asked to choose between five different answer options for each media type: “never”, “approximately 30 min/day”, “between 1 and 2 h/day”, “between 3 and 4 h/day”, “longer than 4 h/day”. For clarity and comparison reasons, the categories “never”, “approximately 30 min/day” and “between 1 and 2 h/day” were summarized into the new category “2 or less than 2 h daily”. The answer options “between 3 and 4 h/day” and “longer than 4 h/day” were summed up to the category “more than 2 h daily”. This categorization made it possible to compare the results to the recommendation to limit daily media consumption times to less than two hours a day [8,9,10]. Furthermore, another dichotomous variable “total screen time” was created. It indicated whether at least one type of media was used more than two hours daily.

The frequencies of physical activity and outdoor activity were assessed by three questions (participation in organized sports in clubs, participation in non-organized sports, playing outside). Children were asked to choose between five different answer categories: “never”, “less often than once/week”, “between 1 and 2 times/week”, “between 3 and 5 times/week”, “nearly every day”. For further analysis, the first three categories were merged into the category “less than 3 times a week”, whereas the answer options “between 3 and 5 times/week” and “nearly every day” were summed up into the category “minimum 3 times a week”. Another created dichotomous variable “total physical activity” indicated whether or not at least one type of physical activity was performed a minimum of three times/week.

Two separate questions indicated engagement in choir/orchestra and theatre/dancing. These questions could be answered by either “yes” or “no”.

Finally, social life was indicated by asking the participants how often they met their friends last week. Answer categories were “never”, “rarely”, “sometimes”, “often” and “always”. For further analysis, the categories “never”, “rarely” and “sometimes” were summed up to the category “never/sometimes” and the categories “often” and “always” were merged to the category “often/always”.

### 2.3. Statistical Analysis

R, version 3.5.2 (R Foundation for Statistical Computing, Vienna, Austria) was used for all statistical analyses. The first analysis, a multiple logistic regression, investigated associations of each leisure activity (as dependent variable) with gender, SES and age (as independent variables). Age and SES were included as continuous measures and gender as two-level categorical variable. Coefficients were reported as odds ratios (ORs).

To examine the time trends of leisure activities of all participants, multiple logistic regressions were used. The date of assessment was the independent variable and the leisure activities the dependent variables. Date of assessment was described as years transformed to decimal numbers (e.g., 2012-07-20 = 2012.55). All associations were adjusted for gender, SES and age, i.e., these variables were included as covariates.

Associations of screen time and physical activity with other leisure activities (outdoor activity, chorus/orchestra, theatre/dancing and social life) were assessed by multiple logistic regression with total screen time or total physical activity as independent variable and the other leisure activities (outdoor activity, chorus/orchestra, theatre/dancing and social life) as dependent variables. Again, all associations were adjusted for gender, SES and age.

Every statistical model was checked for interactions between the independent variables and the control variables age (as continuous variable) and gender. The interaction was included in the final model if the interaction was significant (*p* < 0.05) and the model quality was preserved (indicated by a variance inflation factor < 5).

## 3. Results

### 3.1. Distribution of Leisure Activities and Associations with Gender, Social Class, and Age

The distribution of the different leisure activities can be seen in Table 1. For better comparability, the distributions are presented separately for boys and girls, for two age groups (10- to 13-year-olds and 14- to 18-year-olds) and for the three SES groups (low, middle, and high). The results of the statistical analyses on the associations between leisure activities and gender, SES and age are listed in Table 2.

As can be seen in Table 1, more than half of all participants reported using at least one type of media more than two hours a day. While the difference between boys and girls in their total screen time was not significant (odds ratio (OR) = 1.05, *p* > 0.05), girls used mobile phones significantly more frequently than boys (OR = 1.99, *p* < 0.001). In contrast, game consoles were used more excessively by boys than by girls (OR = 0.14, *p* < 0.001). There was no significant association between the other types of media and gender (all *p* > 0.05). In the total sample, almost one half of the participating children reported being physically active at least three times a week in clubs or non-organized settings and claimed to be active outdoors at least three times a week. Overall, the data demonstrated that boys were more active than girls in total physical activity (OR = 0.50, *p* < 0.001), in organized sports (OR = 0.57, *p* < 0.001), in non-organized sports (OR = 0.54, *p* < 0.001), and in outdoor activities (OR = 0.64, *p* < 0.001). Engagement in choir/orchestra and theater/dancing was reported by approximately one third of all participants. However, the difference between boys and girls was very large. Girls claimed to be a member in a choir/orchestra (OR = 2.72, *p* < 0.001) and in theater/dancing (OR = 3.66, *p* < 0.001) significantly more often than boys. Concerning social life, two third of all participants asserted meeting their friends often/always, with no significant difference between boys and girls (OR = 0.98, *p* > 0.05).

Each type of media was used more extensively by participants with a lower SES. The SES showed significant positive associations with the usage of TV (OR = 0.88, *p* < 0.001), game consoles (OR = 0.87, *p* < 0.001), computer (OR = 0.94, *p* < 0.001), and mobile phone (OR = 0.93, *p* < 0.001), and with the total screen time (OR = 0.90, *p* < 0.001). Moreover, participants with a higher SES were in total more physically active (OR = 1.06, *p* < 0.001), more active in organized sports (OR = 1.05, *p* < 0.01), more engaged in choir/orchestra (OR = 1.07, *p* < 0.001) and theater/dancing (OR = 1.04, *p* < 0.05) and met their friends more often (OR = 1.05, *p* < 0.01) than children with a lower SES. Non-organized physical activity and outdoor activity, in contrast, were not significantly associated with the SES (all *p* > 0.05).

Generally, older adolescents used screen-based media more frequently than younger participants (OR = 1.31, *p* < 0.001). In particular, computers (OR = 1.36, *p* < 0.001) and mobile phones (OR = 1.28, *p* < 0.001) were used significantly more frequently by older than by younger participants. The use of game consoles and TV, however, was not significantly associated with age (all *p* > 0.05); whereas younger children were in total more physically active (OR = 0.94, *p* < 0.05), and more active in organized sports and in outdoor activities (OR = 0.73, *p* < 0.001) than older adolescents. For other leisure activities, no significant association with age could be found (all *p* > 0.05).

### 3.2. Time Trends in Leisure Activities from 2011 to 2017

**Figure 1:**
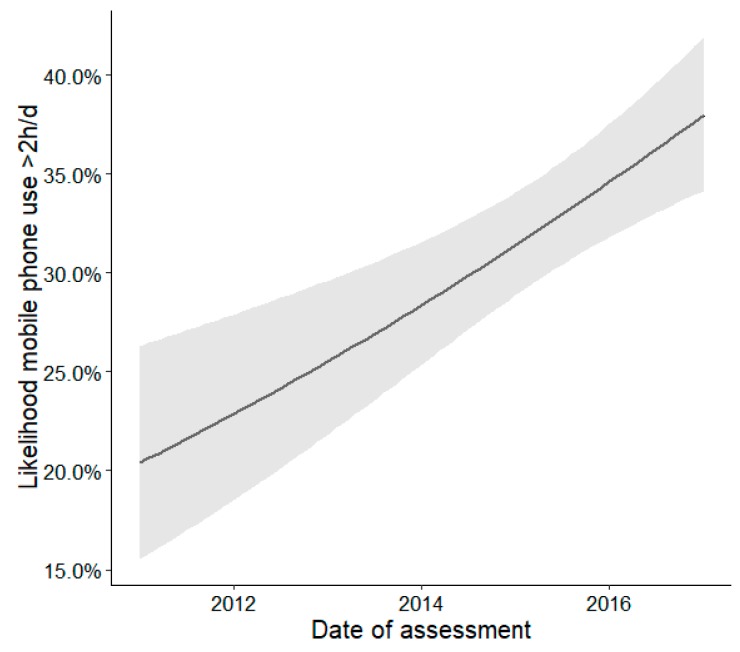
Effects plot illustrating the estimated changes over time (2011–2017) described as association between mobile phone use and date of assessment. The shaded area indicates the 95% confidence intervals of the estimated effect.

Changes over time described as associations between leisure activities and date of assessment are shown in Table 3. The usage of mobile phones increased significantly from 2011 to 2017 (OR = 1.16, *p* < 0.001). As can be seen in Figure 1, in 2011, 20% of children were estimated to show a mobile phone use of more than 2 h/day, compared to more than 35% in 2017. In contrast, there were no significant changes in the usage of the other types of screen-based media and total screen time (all *p* > 0.05).

Participation in organized and non-organized sports, total physical activity, and outdoor activity did not change significantly between 2011 and 2017 (all *p* > 0.05). Similarly, the frequency of meeting friends remained constant over the last years (OR = 1.01, *p* = 0.71).

**Figure 2:**
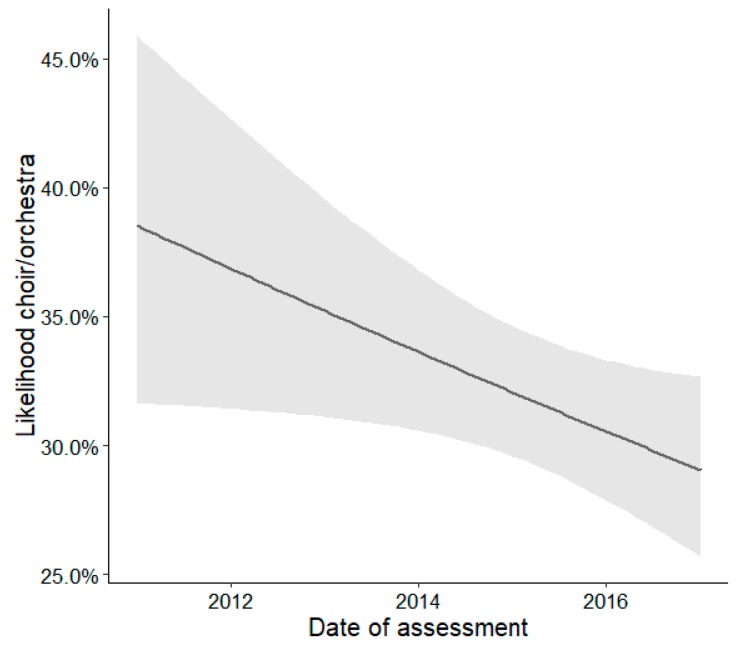
Effects plot illustrating the estimated changes over time (2011–2017) described as association between choir/orchestra and date of assessment. The shaded area indicates the 95% confidence intervals of the estimated effect.

**Figure 3:**
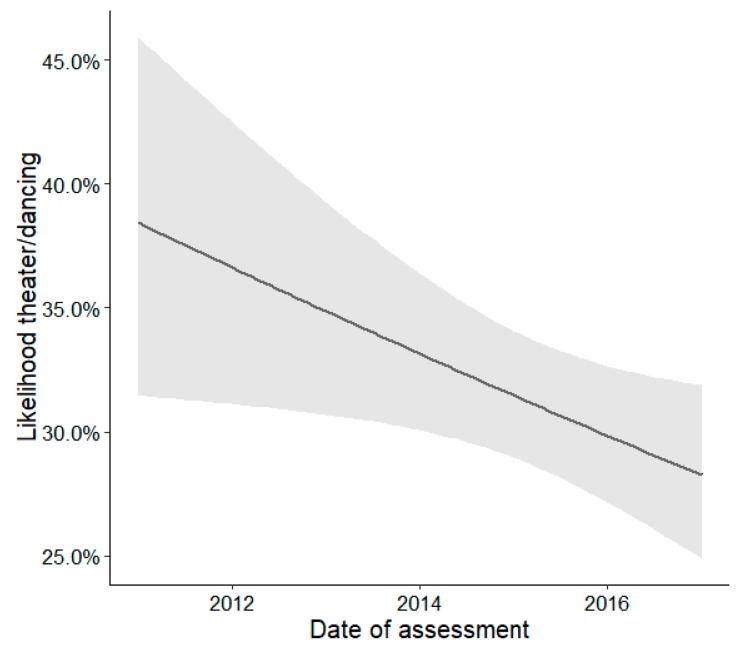
Effects plot illustrating the estimated changes over time (2011–2017) described as association between theater/dancing and date of assessment. The shaded area indicates the 95% confidence intervals of the estimated effect.

In contrast, engagement in choir/orchestra (OR = 0.93, *p* < 0.05, Figure 2.) and theater/dancing (OR = 0.93, *p* < 0.05, Figure 3) decreased between 2011 and 2017.

### 3.3. Associations of Total Screen Time and Total Physical Activity with Other Leisure Activities

The results of the logistic regression analysis for associations of total screen time and total physical activity with other leisure activities are presented in Table 4. Total screen time showed a significant negative association with organized (OR = 0.77, *p* < 0.05), non-organized (OR = 0.69, *p* < 0.01) and total physical activity (OR = 0.65, *p* < 0.001). For children using at least one medium for more than two hours per day, the estimated likelihood to be physically active a minimum of three times a week was 39%. For children showing shorter screen times, the likelihood was 50%. Moreover, total screen time was significantly negatively associated with outdoor activity (OR = 0.64, *p* < 0.001). For the other leisure activities no significant associations were found (all *p* > 0.05).

Total physical activity was significantly positively related to outdoor activity (OR = 2.73, *p* < 0.001). A significant interaction with gender showed that this association was stronger for boys (OR = 3.56, *p* < 0.001) than for girls (OR = 2.08, *p* < 0.001). Beyond this, active children reported meeting their friends more often (OR = 1.63, *p* < 0.001) than less active participants. For children participating in sports a minimum of three times a week, the estimated likelihood to meet friends often was 72%, in contrast to 61% for children being physically active less frequently. A significant interaction with gender showed that this association was only significant for boys (OR = 2.15, *p* < 0.001) but not for girls (OR = 1.20, *p* > 0.05). The usage of the most types of screen-based media, on the other hand, was negatively associated with total physical activity. Children being physically active a minimum of three times a week spent less time watching TV (OR = 0.69, *p* < 0.01), using game consoles (OR = 0.56, *p* < 0.05) and computers (OR = 0.56, *p* < 0.001). However, there was no significant association between total physical activity and mobile phone use (OR = 1.04, *p* > 0.05). A significant interaction with gender showed that the association of total physical activity with computer use was only significant for boys (OR = 0.42, *p* < 0.001) but not for girls (OR = 0.78, *p* > 0.05). Similar to the results regarding the total screen time, choir/orchestra and theater/dancing were not significantly associated with total physical activity (all *p* > 0.05).

## 4. Discussion

The present study examined leisure activities of children and adolescents from the ages of 10 to 18 years. First, the distribution of leisure activities and differences between gender, age groups and social class among healthy children were considered. Furthermore, time trends of leisure activities were explored and associations of total screen time and total physical activity with other leisure activities were assessed.

### 4.1. Distribution of Leisure Activities and Associations with Gender, Social Class, and Age

Overall, the media use of the participating children was high. More than one half exceeded the recommendation of a daily media use of less than two hours a day with just one type of media [8,9]. However, meeting friends, physical and outdoor activity also represent popular leisure behaviors. Approximately half of the participants claimed to be physically active and to play outside a minimum of three times a week. The frequency of meeting friends at least often was even higher. Choir/orchestra and theatre/dancing were slightly less popular, with one third of the participants reporting engagement in these activities.

While older children and adolescents used screen-based media more frequently, they were also less physically active. This finding is in line with other studies [12,17,18]. The frequency of meeting friends and of engaging in choir/orchestra and in theater/dancing did not differ significantly between younger and older children, suggesting that social life and the interest in artistic leisure activities do not change during development.

As already reported in other studies [12,13,16,35], children and adolescents from families with lower SES used screen-based media more often and were physically less active than children from higher social classes. Moreover, children from a higher social class were also more frequently involved in choir/orchestra, theater/dancing and meeting their friends. In contrast, a nationwide survey on the leisure behavior of German children and adolescents showed that children with lower SES met their friends more often [22]. This discrepancy suggests that the relationship between SES and social life is far from being understood. Methodological differences between studies might also play a role. In the nationwide survey [22], SES was indicated by school type, not by the education or income of parents.

With respect to gender differences, girls used mobile phones more often, while boys played more console games. This is comparable to previous findings [7,11]. Furthermore, boys were more physically active than girls. These results correspond to the current state of research [13,16]. A large gender difference consisted in the engagement in choir/orchestra and theater/dancing with girls being more engaged than boys. This finding is in line with data from the Health Behaviour in School-Aged Children (HBSC) study [19]. With respect to social life (meeting friends), no essential difference between girls and boys could be shown. This finding is not in line with other previous studies in which boys were more likely to go out with friends [22]. This result is not in line with the findings of a nationwide survey in which boys were more likely to go out with friends than girls [22]. However, in the survey, only descriptive results were presented, with no verification by statistical analyses.

Overall, the results underline the need to inform families and schools about the importance of limiting media usage times and promoting physical activity, especially in older children, girls, and children from lower social strata.

### 4.2. Trends in Leisure Activities from 2011 to 2017

The data showed that the use of mobile phones increased drastically between 2011 and 2017, whereas there was no significant difference in the usage of the other types of screen-based media. This is only partly in line with previous studies showing a general increase of media use [6,7], especially mobile media use (including mobile phones but also computers), but a decline of television [5]. These differences can be explained in part by the way the answer categories were summed up and by the period of time the data was collected. The studies mentioned were carried out several years ago when the newer media did not yet play a major role.

Physical activity did not change significantly in the years from 2011 to 2017. This is consistent with previous findings [5,15]. Moreover, outdoor activity and social life (meeting friends) have remained almost unchanged. In contrast, engagement in choir/orchestra and theater/dancing decreased between 2011 and 2017.

These results show an alarming development with an increase in the importance of the newer media, especially the use of mobile phones, but a decline of leisure activities related to arts and music.

### 4.3. Associations of Total Screen Time and Total Physical Activity with Other Leisure Activities

Participants with higher screen times (especially regarding TV and using games consoles) were physically active and played outdoors less frequently than children with lower screen times. This is in good agreement with some previous studies [23,24,26], but contradicts other studies which did not find any associations between media use and physical activity [25,26,27]. Our results suggest that high media usage might displace more active leisure behavior and hinder children from spending time outdoors. Furthermore, our results suggest that male computer users show a higher risk to neglect active behaviors than female computer users.

In contrast to screen time, physical activity was positively associated with playing outdoors. This association might be explained by the fact that sports might represent an outdoor activity. Furthermore, physically active boys (but not girls) could be shown to meet their friends more frequently than less active boys. Boys might meet their friends with the purpose of doing sports together. This assumption is in line with a previous study showing that children within a peer group of physically active friends are also more active themselves [28].

Interestingly, the engagement in chorus/orchestra and theatre/dancing was related to neither screen time nor physical activity. This suggests that media use and physical activity do not distract leisure activities related to music and arts. However, we did not assess the frequency of these activities. The engagement in choir/orchestra or theatre/dancing might also be motivated by children’s parents rather than children themselves. This could further explain the independence of behavior that is performed in a more self-determined way (media use or physical activity).

### 4.4. Strengths and Limitations

The strengths of the present studies are the large sample size and the variety of leisure behaviors considered.

Nonetheless, some limitations have to be noticed. This study did not differentiate between leisure activities performed on weekdays vs. weekends. Children have more free time during weekends than during weekdays, and the distribution of leisure activities might differ.

In addition, our questionnaires interrogated about physical activity per week. For better comparability with the literature and the recommendation of the World Health Organization (WHO) it would be better to consider the daily physical activity [36] and to distinguish between different forms of physical activity (moderate, vigorous etc.).

Finally, the data were based on self-reports. However, young children (e.g., 10-year-olds) in particular might have a distorted time perception and, therefore, have difficulties judging the real time they spend on different leisure activities.

## 5. Conclusions

The present study underlines the increasing importance of screen-based media in the lives of children and adolescents and suggests that a high media use may displace more active leisure behaviors. The use of mobile phones by girls and the generally high media use of children from lower social strata, who are also significantly less physically active, are particularly worth mentioning. Since the overall use of mobile phones has also increased drastically between 2011 and 2017, it is important to continue monitoring this development. The study findings, furthermore, indicate a decline in leisure activities related to arts and music. In contrast, social contacts and physical activity remained unchanged and could be shown to interact positively. The findings imply that health-conscious behavior (sufficient physical and outdoor activity, limited screen times) should be promoted at an early stage of child development in order to avoid the creeping in of harmful habits with consequences on leisure activities in adulthood.

## Figures and Tables

**Table 1:** Distribution of leisure activities among healthy children in the LIFE Child study cohort (*N* = 1449).

Leisure Activity		All (*N* = 1449)	Gender		SES Group			Age Group	
			Male (*N* = 717)	Female (*N* = 732)	Low (*N* = 215)	Middle (*N* = 861)	High (*N* = 370)	10–13 Years (*N* = 694)	14–18 Years (*N* = 755)
TV ^a^	*N* (%)	300 (20.7%)	154 (21.5%)	146 (20.0%)	77 (35.8%)	174 (20.2%)	49 (13.2%)	134 (19.3%)	166 (22.0%)
Game console ^a^	*N* (%)	97 (6.7%)	84 (11.7%)	14 (1.9%)	26 (12.1%)	59 (6.9%)	13 (3.5%)	49 (7.1%)	49 (6.5%)
Computer ^a^	*N* (%)	403 (27.8%)	203 (28.3%)	200 (27.3%)	74 (34.4%)	251 (29.2%)	77 (20.8%)	109 (15.7%)	294 (38.9%)
Mobile phone ^a^	*N* (%)	493 (34.0%)	187 (26.1%)	306 (41.8%)	92 (42.8%)	294 (34.1%)	107 (28.9%)	157 (22.6%)	336 (44.5%)
Total screen time ^b^	*N* (%)	781 (53.9%)	376 (52.4%)	405 (55.3%)	150 (69.8%)	464 (53.9%)	166 (44.9%)	282 (40.6%)	499 (66.1%)
Sports organized (in clubs) ^c^	*N* (%)	353 (24.4%)	213 (29.7%)	140 (19.1%)	32 (14.9%)	212 (24.6%)	109 (29.5%)	179 (25.8%)	174 (23.0%)
Sports non-organized ^c^	*N* (%)	409 (28.2%)	249 (34.7%)	161 (22.0%)	58 (27.0%)	233 (27.1%)	117 (31.6%)	218 (31.4%)	192 (25.4%)
Total physical activity ^d^	*N* (%)	639 (44.1%)	379 (52.9%)	260 (35.5%)	76 (35.3%)	370 (43.0%)	191 (51.6%)	324 (46.7%)	315 (41.7%)
Outdoor activity ^c^	*N* (%)	745 (51.4%)	413 (57.6%)	333 (45.5%)	106 (49.3%)	431 (50.1%)	206 (55.7%)	450 (64.8%)	296 (39.2%)
Choir/orchestra ^e^	*N* (%)	477 (33.0%)	159 (22.2%)	318 (45.5%)	57 (26.5%)	276 (32.1%)	143 (38.6%)	218 (31.4%)	259 (34.3%)
Theater/dancing ^e^	*N* (%)	476 (32.9%)	137 (19.1%)	339 (46.3%)	65 (30.2%)	276 (32.1%)	134 (36.2%)	202 (29.1%)	274 (36.3%)
Meeting friends ^f^	*N* (%)	956 (66.0%)	475 (66.2%)	481 (65.7%)	122 (56.7%)	570 (66.2%)	261 (70.5%)	453 (65.3%)	503 (66.6%)

^a^ more than two hours a day; ^b^ at least one type of media more than two hours a day; ^c^ a minimum of three times a week; ^d^ organized or non-organized sports a minimum of three times a week; ^e^ yes; ^f^ often/always; SES = socio-economic status.

**Table 2:** Associations between leisure activities and gender, SES and age (*N* = 1449).

Leisure Activity	Gender ^g^	SES	Age
	OR (95% CI)	OR (95% CI)	OR (95% CI)
TV ^a^	0.90 (0.69–1.16)	0.88 (0.85–0.91) ***	1.01 (0.95–1.07)
Game console ^a^	0.14 (0.08–0.25) ***	0.87 (0.81–0.92) ***	0.99 (0.90–1.07)
Computer ^a^	0.86 (0.67–1.09)	0.94 (0.91–0.97) ***	1.36 (1.28–1.44) ***
Mobile phone ^a^	1.99 (1.58–2.51) ***	0.93 (0.90–0.96) ***	1.28 (1.22–1.36) ***
Total screen time ^b^	1.05 (0.84–1.31)	0.90 (0.87–0.93) ***	1.31 (1.24–1.37) ***
Sports organized (in clubs) ^c^	0.57 (0.44–0.72) ***	1.05 (1.02–1.09) **	0.95 (0.90–1.00)
Sports non-organized ^c^	0.54 (0.43–0.68) ***	1.02 (0.99–1.06)	0.92 (0.88–0.97) **
Total physical activity ^d^	0.50 (0.40–0.61) ***	1.06 (1.03–1.09) ***	0.94 (0.90–1.00) *
Outdoor activity ^c^	0.64 (0.51–0.80) ***	1.03 (1.00–1.06)	0.73 (0.69–0.77) ***
Choir/orchestra ^e^	2.72 (2.16–3.42) ***	1.07 (1.04–1.10) ***	1.03 (0.98–1.08)
Theater/dancing ^e^	3.66 (2.88–4.63) ***	1.04 (1.01–1.08) *	1.03 (0.98–1.08)
Meeting friends ^f^	0.98 (0.79–1.22)	1.05 (1.02–1.08) **	1.01 (0.96–1.06)

^a^ more than two hours a day; ^b^ at least one type of media more than two hours a day; ^c^ a minimum of three times a week; ^d^ organized or non-organized sports a minimum of three times a week; ^e^ yes; ^f^ often/always; ^g^ reference: Male; CI = Confidence Interval; OR = Odds Ratio; * *p* < 0.05, ** *p* < 0.01, *** *p* < 0.001.

**Table 3:** Changes over time (2011–2017) described as associations between leisure activities and date of assessment (*N* = 1449).

Leisure Activity	Association with Date of Assessment
	OR (95% CI)
TV	1.02 (0.94–1.10)
Game console	1.00 (0.88–1.09)
Computer	0.97 (0.90–1.04)
Mobile phone	1.16 (1.08–1.24) ***
Total screen time	1.00 (0.94–1.07)
Sports organized (in clubs)	1.03 (0.96–1.11)
Sports non-organized	1.03 (0.96–1.10)
Total physical activity	1.05 (0.99–1.12)
Outdoor activity	0.94 (0.88–1.01)
Choir/orchestra	0.93 (0.87–1.00) *
Theater/dancing	0.93 (0.86–0.99) *
Meeting friends	1.01 (0.95–1.08)

OR = Odds Ratio; * *p* < 0.05, *** *p* < 0.001. All associations are adjusted for age, gender and socio-economic status (SES).

**Table 4:** Associations of total screen time and total physical activity with other leisure activities (*N* = 1449).

Leisure Activity	Association with Total Screen Time	Association with Total Physical Activity
	OR (95% CI)	OR (95% CI)
Sports organized (in clubs)	0.77 (0.60–1.00) *	
Sports non-organized	0.69 (0.54–0.88) **	
Total physical activity	0.65 (0.52–0.81) ***	
Outdoor activity	0.64 (0.51–0.80) ***	2.73 (2.17–3.45) ***
Choir/orchestra	0.87 (0.68–1.11)	0.98 (0.78–1.24)
Theater/dancing	1.10 (0.86–1.41)	1.05 (0.83–1.33)
Meeting friends	0.99 (0.78–1.25)	1.63 (1.29–2.04) ***
TV		0.69 (0.52–0.91) **
Game console		0.56 (0.36–0.88) *
Computer		0.56 (0.44–0.73) ***
Mobile phone		1.04 (0.82–1.31)

OR = Odds Ratio; * *p* < 0.05; ** *p* < 0.01; *** *p* < 0.001. All associations are adjusted for age, gender, socio-economic status (SES).
